# BMP4 dose dictates lineage specification bias in human periodontal ligament stem cells

**DOI:** 10.3389/fbioe.2025.1738051

**Published:** 2026-01-06

**Authors:** Yihong Li, Qinghuang Tang, Liwen Li, Chensheng Lin, Minhui Guo, Yanding Zhang, Fuhua Yan, Minkui Lin

**Affiliations:** 1 Clinical Research Center for Oral Tissue Deficiency Diseases of Fujian Province and Fujian Key Laboratory of Oral Diseases and Fujian Provincial Engineering Research Center of Oral Biomaterial, School and Hospital of Stomatology, Fujian Medical University, Fuzhou, China; 2 Institute of Stomatology and Laboratory of Oral Tissue Engineering, School and Hospital of Stomatology, Fujian Medical University, Fuzhou, Fujian, China; 3 Department of Cell and Molecular Biology, Tulane University, New Orleans, LA, United States; 4 Department of Biological Sciences, College of Arts and Sciences, FOXG1 Research Center, University at Buffalo, Buffalo, NY, United States; 5 Fujian Key Laboratory of Developmental and Neural Biology and Southern Center for Biomedical Research, College of Life Sciences, Fujian Normal University, Fuzhou, Fujian, China; 6 Department of periodontology, Nanjing Stomatological Hospital, Affiliated Hospital of Medical School, Institute of Stomatology, Nanjing University, Nanjing, China

**Keywords:** BMP4, osteogenic and tenogenic differentiation, PDLSC, RUNX2, SCX

## Abstract

**Introduction:**

Periodontal ligament stem cells (PDLSCs) represent a promising therapeutic cell source for regenerating periodontal tissues impaired by disease. While exogenous BMP4 has been used to induce human PDLSC differentiation into distinct periodontal cell lineages, its dose-dependent effects on lineage specification remain poorly understood.

**Methods:**

Here, we systematically investigated how BMP4 dosage modulates human PDLSC differentiation toward osteogenic and tenogenic lineages using single-cell RNA sequencing and *in vitro* functional assays.

**Results:**

Single-cell transcriptomic analysis revealed that endogenous *BMP4* expression inversely correlated with *RUNX2* but positively correlated with *SCX* within PDLSC subpopulations. Pseudotemporal analysis demonstrated biphasic differentiation dynamics: low *BMP4* expression corresponded to high expression of both markers, while *BMP4* upregulation inversely correlated with *RUNX2* but positively correlated with *SCX*. *In vitro* validation confirmed dose-dependent effects: low-dose BMP4 (10 ng/mL) sustained multipotency by upregulating both *RUNX2* and *SCX*, while high-dose BMP4 (100 ng/mL) promoted tenogenic differentiation and suppressed osteogenic commitment. BMP4 overexpression induced nuclear SCX accumulation, confirming its pivotal role in tenogenic lineage commitment. Mechanistically, osteogenic differentiation required both BMPR1A and BMPR1B receptors, whereas tenogenic differentiation depended exclusively on BMPR1B signaling.

**Conclusion:**

These findings demonstrate that precise BMP4 dosage regulation dictates PDLSC differentiation outcomes, providing crucial insights for optimizing PDLSC-based periodontal regenerative therapies.

## 1 Introduction

Periodontal diseases represent a major oral health problem, affecting 20%–50% of the worldwide population and causing progressive destruction of periodontal tissues—including the periodontal ligament (PDL), cementum, and alveolar bone—that support and maintain teeth (Bharuka and Reche, 2024; Chen et al., 2022). Periodontitis, the most severe form of periodontal disease, is the leading cause of tooth loss in adults worldwide (Xie et al., 2025). This widespread prevalence has driven intensive research into regenerative therapeutic approaches aimed at restoring damaged periodontal tissues and their essential functions.

Successful periodontal regeneration requires the coordinated integration of three fundamental components: morphogenetic signaling molecules, responsive stem/progenitor cells, and appropriate extracellular matrix scaffolds (Meng et al., 2022; Ouchi and Nakagawa, 2020). Among potential stem cell sources, periodontal ligament stem cells (PDLSCs) have emerged as particularly promising candidates for regenerative applications (Morita et al., 2025). PDLSCs demonstrate remarkable multipotency under defined culture conditions. They can differentiate into several types of mature cells, including osteoblasts and chondrocytes, which are key components for repairing damaged periodontal tissues (Ye et al., 2014; Yuda et al., 2015; Menicanin et al., 2014). Importantly, when transplanted, PDLSCs can regenerate cementum and PDL-like structures (Seo et al., 2004), making them ideal therapeutic candidates for periodontal tissue regeneration (Bharuka and Reche, 2024; Ouchi and Nakagawa, 2020).

Understanding how to precisely control PDLSC differentiation toward specific lineages remains crucial for optimizing periodontal regeneration strategies (Hughes et al., 2010; Rodriguez-Lozano et al., 2012). Bone morphogenetic protein 4 (BMP4), a secreted glycoprotein belonging to the transforming growth factor-β superfamily, has demonstrated significant potential in regulating stem cell fate decisions (Jain et al., 2013). BMP4 signals through a well-characterized pathway involving binding to type I (BMPRI) and type II (BMPRII) receptor kinases, leading to phosphorylation and activation of receptor-regulated Smads (Smad1/5/8), which subsequently form complexes with Smad4 to regulate target gene expression (Massague et al., 2005; Datto and Wang, 2000; Feng and Derynck, 2005). Additionally, BMP4 also activates signaling via a non-canonical Smad-independent pathways. Exogenous BMP4 has been widely used to induce differentiation of mesenchymal stem cells and pluripotent stem cells into different type of cells *in vitro* (Beederman et al., 2013; Kamarudin et al., 2018) and to promote regeneration of multiple tissues, including teeth, bone, cartilage and periodontal tissue (Pan et al., 2023; Anusuya et al., 2016). *In vitro* differentiation studies using exogenous BMP4 at concentrations ranging from 10 ng/mL to 100 ng/mL demonstrate dose-dependent effects on embryonic stem cells fate determination (Setiawan et al., 2022). However, little is understood about how BMP4 signaling pathways drive *in vitro* hPDLSC differentiation toward certain cell lineages.

While BMP4’s role in maintaining PDLSC stemness and promoting osteogenic differentiation has been established (Beederman et al., 2013; Liu et al., 2013), its effects on tenogenic lineage commitment remain poorly understood. Given the critical importance of both osteogenic and tenogenic differentiation in periodontal tissue regeneration, elucidating BMP4’s dose-dependent effects on these divergent lineages is essential. Recent advances in single-cell RNA sequencing technology have provided unprecedented opportunities to dissect the molecular mechanisms underlying stem cell differentiation at cellular resolution (Pijuan-Sala et al., 2018), offering new insights into the heterogeneity and developmental plasticity of PDLSC populations. In this study, we investigated the dose-dependent effects of BMP4 on hPDLSC differentiation, with particular focus on both osteogenic and tenogenic differentiation, using Runt-Related Transcription Factor 2 (RUNX2) and Scleraxis BHLH Transcription Factor (SCX) as indicators of osteogenic and tenogenic commitment (Komori, 2010; Schweitzer et al., 2001), respectively. Through a combination of comprehensive single-cell transcriptomic analyses and *in vitro* functional assays, we demonstrate that BMP4 elicits dose-dependent responses in hPDLSCs, promoting tenogenic differentiation at higher concentrations while maintaining multipotency at lower concentrations. Our findings reveal the molecular mechanisms underlying these differential responses, providing a foundation for developing more precise strategies to control PDLSC differentiation in periodontal regenerative therapies.

## 2 Results

### 2.1 Single-cell transcriptomic analysis reveals heterogeneity and BMP4-mediated lineage bias in hPDLSCs

**FIGURE 1:**
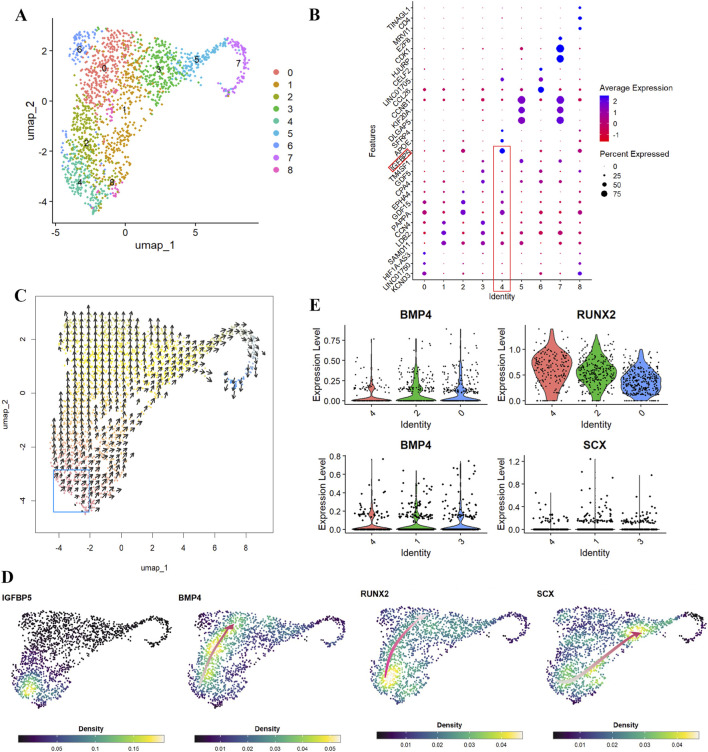
Single-cell RNA-seq analyses reveal BMP4, RUNX2 and SCX gene expression dynamics during human periodontal ligament stem cells (hPDLSCs) differentiation. **(A)** Uniform manifold approximation and projection (UMAP) visualization of nine subpopulations (clusters 0–8) in hPDLSCs. **(B)** Dot plots showing expression profiles of the top 3 markers across seven subpopulations. **(C)** VECTOR analysis reveals developmental trajectories of hPDLSCs starting from cluster 4. **(D)** Kernel density estimation shows the expression density of *IGFBP5*, *BMP4*, *RUNX2*, and *SCX* during hPDLSC differentiation. Red lines indicate gene expression trends along the hPDLSC differentiation trajectory, with light red representing lower expression density and dark red representing high expression density. **(E)** Violin plots demonstrate that along hPDLSC differentiation trajectories, *RUNX2* expression decreases following increased *BMP4* expression (cluster 4→2→0), while *SCX* expression increases (cluster 4→1→3).

Human periodontal ligament stem cells (hPDLSCs) demonstrate multilineage differentiation capacity both *in vivo* and *in vitro*, forming cementum/PDL-like tissues *in vivo* and differentiating into osteoblasts, chondrocytes, and adipocytes *in vitro* (Seo et al., 2004; Gay et al., 2007). To gain insight into the multilineage differentiation capacity of hPDLSCs, we analyzed a publicly available single-cell RNA-sequencing (scRNA-seq) dataset from pooled periodontal ligament tissues of third molar obtained from 3 healthy individuals (aged 21–27 years, 1 female and 2 males) (Yang et al., 2024). Unsupervised clustering using Seurat (Hao et al., 2024) identified nine distinct cell subpopulations (Figure 1A, clusters 0–8), each exhibiting unique gene expression patterns (Figure 1B) that revealed cellular heterogeneity within hPDLSCs.

Notably, cluster 4 was characterized by expression of *IGFBP5* (Figure 1B, red rectangles), a stem cell marker for dental mesenchymal progenitors that contribute specifically to periodontal cell lineages (Seidel et al., 2017). Consistent with this finding, unsupervised inference of developmental directions using VECTOR (Zhang et al., 2020) revealed that all subpopulations derived from *IGFBP5*-expressing cluster 4 cells (Figure 1C, blue rectangle) differentiating along two major developmental directions. Given the observed developmental heterogeneity within hPDLSCs, we then investigated the expression patterns of key regulatory factors governing lineage specification. RUNX2 is the master transcription factor required for osteogenic lineage determination (Komori, 2010), while SCX serves as the key transcription factor for tenogenic differentiation and is highly specific for tendon and ligament tissues (Schweitzer et al., 2001). Since osteogenic and tenogenic differentiation represent critical developmental processes for periodontal tissue regeneration, we analyzed the expression of these lineage-specific transcription factors alongside BMP4, which is widely used to induce *in vitro* differentiation of human mesenchymal stem cells (Setiawan et al., 2022). Kernel gene-weighted density estimation revealed that within *IGFBP5*-expressing cluster 4 cells, higher *BMP4* expression correlated with higher *RUNX2* expression, while lower BMP4 expression correlated with higher *SCX* expression (Figure 1D), consistent with the established osteogenic function of BMP4 (Beederman et al., 2013). Interestingly, along the two major developmental directions, *BMP4* upregulation correlated with decreased *RUNX2* and increased *SCX* expression (Figure 1D, red arrows), implicating a BMP4 dose-dependent effect on hPDLSC lineage specification. Quantitative analysis confirmed that *BMP4* upregulation inversely correlated with *RUNX2* (Figure 1E, cluster 4→2→0) and positively correlated with SCX expression (Figure 1E, cluster 4→1→3). These results uncover previously unrecognized levels of cellular heterogeneity and developmental plasticity within hPDLSCs and indicate that BMP4 regulates the bias between osteogenic and tenogenic differentiation programs in a dose-dependent manner.

### 2.2 Pseudotemporal trajectory analysis uncovers biphasic expression dynamics of BMP4, RUNX2 and SCX during hPDLSC lineage specification

**FIGURE 2:**
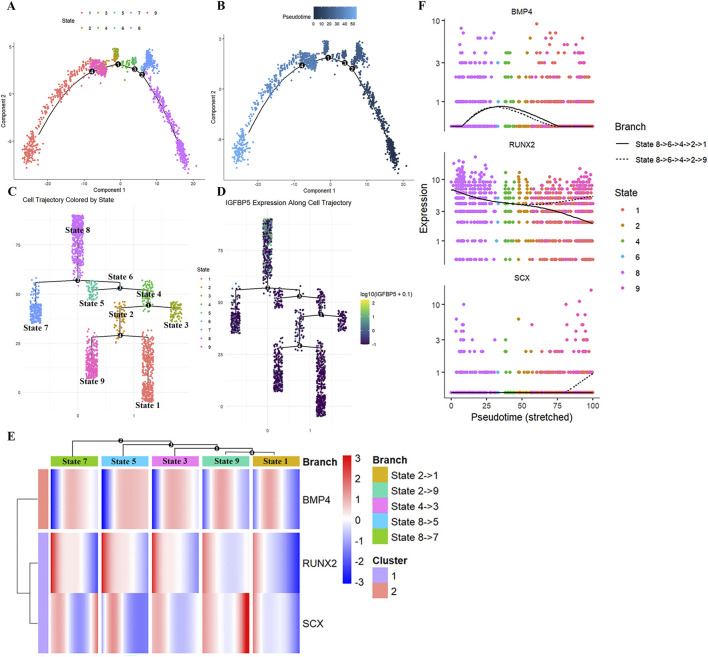
Dynamics of *BMP4*, *RUNX2* and *SCX* expression during pseudotemporal differentiation of hPDLSCs. **(A,B)** Pseudotime analysis illustrating 9 different cellular states **(A)** during hPDLSC differentiation **(B)**. **(C,D)** Multiple branch plots showing that the 9 different cellular states **(C)** originated from State 8 cells expressing *IGFBP5* **(D)**. **(E)** Multiway branched heatmap showing the expression dynamics of *BMP4*, *RUNX2* and *SCX* during hPDLSC differentiation. **(F)** Branching plots demonstrate the bifurcation of *BMP4*, *RUNX2* and *SCX* in different cellular states during hPDLSC differentiation.

To further dissect the temporal dynamics of BMP4, RUNX2 and SCX during hPDLSC lineage specification, we reconstructed the differentiation trajectory using Monocle2 (Qiu et al., 2017). Trajectory analysis identified nine distinct cellular states (Figure 2A, State 1–9) that span four branch points (Figure 2B, number 1–4) over pseudotemporal differentiation of hPDLSCs. Originating from initial state 8, which expresses IGFBP5 (Figure 2D), hPDLSCs bifurcate into five terminal states (Figure 2C, States 7, 5, 3, 9, and 1). Analysis of transition relationships among cell subpopulations (clusters 0–8) confirmed that all subpopulations are derived from cluster 4 cells (Supplementary Figure S1A,C). Cluster 4 cells are defined by IGFBP5 expression (Supplementary Figure S1B), which aligns with the VECTOR inference results (Figure 1C). Notably, BMP4 was expressed throughout the entire hPDLSC differentiation trajectory (Supplementary Figure S1B). Characterization of BMP4, RUNX2 and SCX expression during hPDLSC differentiation revealed a consistent pattern: at the onset of each cellular state transition, BMP4 expression was low, coinciding with high expression of both RUNX2 and SCX (Figure 2E). During subsequent state transitions, BMP4 gradually increased then decreased at all branch points except the bifurcation from state 8 to state 5 (Figure 2E, State 8→5), where BMP4 maintained high expression (Figure 2E, branch point 3). Notably, following upregulation of BMP4 expression, RUNX2 expression immediately decreased at all branch points (Figure 2E). In contrast, SCX expression immediately increased and remained elevated longer than RUNX2 at all branch points except the bifurcation from state 2 to state 1 (Figure 2E, State 2→1). Interestingly, SCX expression peaked at two specific bifurcations: from state 8 to state 7 (Figure 2E, State 8→7) and from state 2 to state 9 (Figure 2E, State 2→9). These peaks occurred after BMP4 expression had declined. Visualization of BMP4, RUNX2, and SCX expression kinetics during hPDLSC cellular state transitions (Figure 2F) further confirmed these observations. Collectively, these results reveal biphasic expression dynamics of BMP4, RUNX2, and SCX expression during hPDLSC differentiation: low BMP4 expression correlates with high co-expression of RUNX2 and SCX, while BMP4 upregulation exhibited an inverse correlation with RUNX2 expression and a positive, dynamic relationship with SCX expression. These findings further support a dose-dependent regulatory mechanism of BMP4 in hPDLSC lineage specification.

### 2.3 BMP4 exerts dose-dependent effects on hPDLSC lineage specification

**FIGURE 3:**
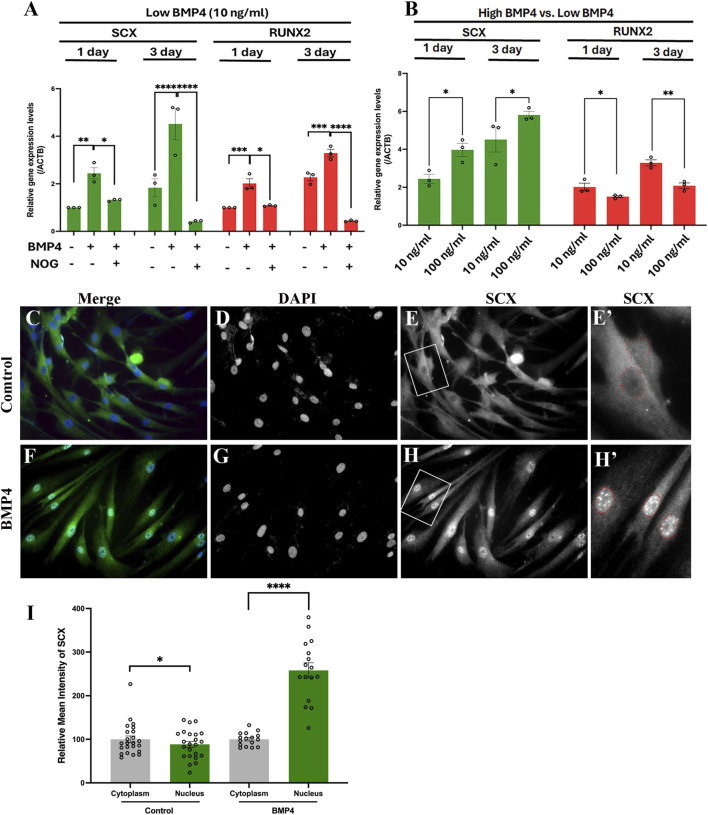
BMP4 induces dose-dependent osteogenic and tenogenic differentiation of hPDLSCs. **(A)** Low-dose BMP4 (10 ng/mL) treatment of hPDLSCs significantly upregulated both *SCX and RUNX2* mRNA expression after 1 day or 3 days, while NOG treatment (1 ng/mL) attenuated upregulation of both genes. **(B)** High-dose BMP4 (100 ng/mL) further enhanced *SCX* upregulation but decreased *RUNX2* expression compared to low-dose BMP4 treatment. **(C–H′)** hPDLSCs treated with control or BMP4 lentivirus for 3 days hPDLSCs with BMP4 overexpression showed increased nuclear SCX accumulation **(F–H′)** compared to control **(C–E′)**. Merged images show SCX and DAPI colocalization **(C,F)**. DAPI labels nuclei **(D,G)**. Cytoplasmic and nuclear SCX distribution in control **(E)** and BMP4-overexpressed cells **(H)** are shown in high-magnification images **(E′,H′)**. **(I)** CellProfiler quantification of SCX fluorescence intensity in nucleus (green) and cytoplasm (gray) demonstrates that BMP4 overexpression significantly increases nuclear SCX accumulation compared to control. Scale bars: 25 μm **(C–H)**, 5 μm **(E′,H′)**; *, *P* ≤ 0.01; ****, *P* ≤ 0.0001 (two-way ANOVA); ns, not significant (*P* > 0.05).

Building on our findings (Figures 1, 2) and previous studies showing that BMPs at various concentrations differently modulate the proliferation and osteogenic differentiation of hPDLSCs (Hakki et al., 2014), we further characterized the osteogenic and tenogenic differentiation potential of hPDLSCs following treatment with low (10 ng/mL) or high (100 ng/mL) BMP4 concentrations. We selected these specific concentrations based on established literature demonstrating that 10 ng/mL represents a threshold for maintaining PDLSC multipotency (Liu et al., 2013), while 100 ng/mL induces robust differentiation responses (Hyun et al., 2017). These concentrations at opposite ends of the physiologically relevant range allowed us to directly test whether BMP4 exerts dose-dependent effects consistent with the biphasic expression patterns observed in our scRNA-seq analysis. Notably, low-concentration BMP4 treatment increased expression of both SCX and RUNX2 (Figure 3A), consistent with our pseudotemporal analysis (Figure 2E). To confirm that BMP4 directly mediates upregulation of these transcription factors, we treated hPDLSCs with Noggin, a well-known BMP4 antagonist. This intervention effectively attenuated the BMP4-induced upregulation of both SCX and RUNX2 (Figure 3A). A key observation emerged when comparing the effects of low- and high-concentration BMP4: relative to the low-dose group, high concentration BMP4 further enhanced SCX expression while simultaneously suppressing RUNX2 expression, which aligns with our scRNA-seq analysis (Figures 1, 2). Given the positive correlation between SCX expression and both exogenous BMP4 treatment (Figure 3B) and endogenous BMP4 expression levels (Figures 1, 2) in hPDLSCs, we next investigated whether SCX responds directly to elevated BMP4. Using a lentiviral overexpression system to upregulate BMP4 in hPDLSCs, we found that BMP4 overexpression increased nuclear accumulation of SCX (Figures 3F–H') by approximately 2.5-fold (Figure 3I) compared with the control group (Figures 3C–E'), directly confirming BMP4’s pivotal role in driving hPDLSCs commitment to the tenogenic lineage. Collectively, these data validate that BMP4 elicits dose-dependent effects on hPDLSC lineage specification: low BMP4 concentration preserves hPDLSC multipotency, whereas high BMP4 concentration promotes tenogenic lineage commitment while attenuating osteogenic differentiation.

### 2.4 Distinct BMP receptor pairs mediate BMP4-dependent osteogenic and tenogenic differentiation of hPDLSCs

**FIGURE 4:**
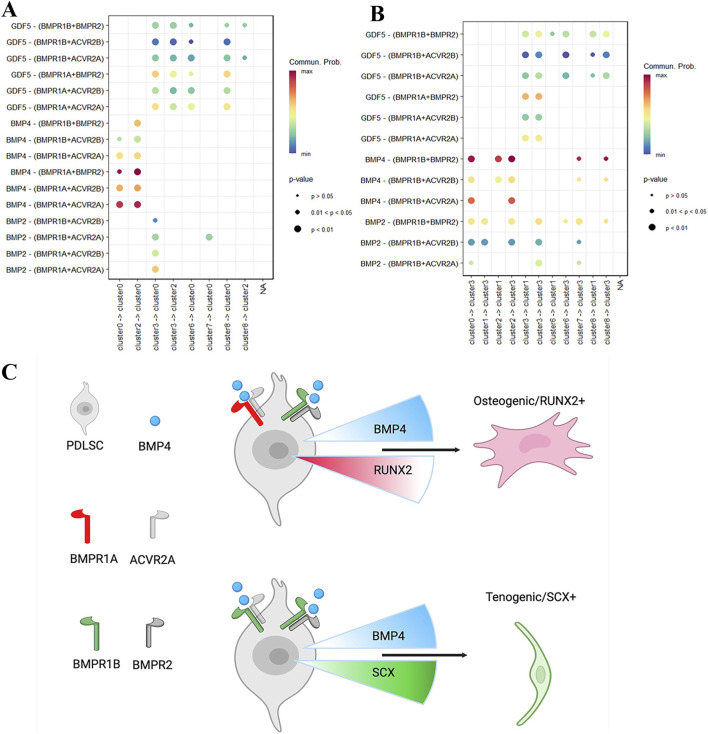
Cell subpopulation-distinct ligand-receptor interactions of BMP signaling in hPDLSCs. **(A,B)** Dot plots demonstrate different ligand-receptor interaction probabilities between RUNX2 expressing (A, Cluster 4 →2 →0) and SCX expressing (B, Cluster 4→1→3) cell subpopulations. **(C)** Diagram showing potential mechanisms underlying dose-dependent osteogenic and tenogenic differentiation of hPDLSCs in response to BMP4 treatment. Differences between osteogenic (RUNX2+) and tenogenic (SCX+) hPDLSCs—distinct receptor pairs (BMPR1A or BMPR1B with type II receptors *versus* BMPR1B exclusively with type II receptors) and differential *RUNX2* (decreasing, red) and *SCX* (increasing, green) expression responses to BMP4 upregulation (blue)—drive diverse differentiation outcomes. The diagram was created with BioRender.com.

Given that BMP4 initiates signal transduction through ligand-receptor interactions, we utilized CellChat (Jin et al., 2021) to infer and analyze the cell-cell communications among hPDLSCs subpopulations (cluster 0–8). This analysis aimed to understand how *in vitro* treatment with high-concentration BMP4 elicits opposing osteogenic and tenogenic differentiation responses (Figure 3). Quantitative assessment of incoming BMP signaling in osteogenic subpopulations (Figure 4A, cluster 0 and 2) and tenogenic subpopulations (Figure 4B, cluster 1 and 3) revealed that BMP4 signaling for these two lineages is transduced through different receptor pairs. In osteogenic hPDLSCs, BMP4 signals are transduced through pairs of BMPR1A or BMPR1B with one of three type II receptors: BMPR2, ACVR2A, and ACVR2B (Figure 4A).

These three type II receptors are functionally similar, as they interact with an overlapping group of TGF-β family growth factors (Chu et al., 2022). In contrast, tenogenic hPDLSCs utilize BMPR1B paired with the same three type II receptors (BMPR2, ACVR2A, and ACVR2B) for BMP4 signaling transduction (Figure 4B). Based on these findings, we propose a mechanistic model underlying the dose-dependent osteogenic and tenogenic differentiation of hPDLSCs in response to *in vitro* BMP4 treatment. Specifically, two key differences between osteogenic (RUNX2+) and tenogenic (SCX+) hPDLSCs collectively drive their divergent differentiation responses: (1) distinct cell surface BMP receptor pairs, and (2) inherently different patterns of RUNX2 and SCX expression changes upon BMP4 upregulation (Figure 4C).

## 3 Discussion

BMP4, a key member of the transforming growth factor-β (TGF-β) superfamily, plays a pivotal role in stem cell lineage commitment and is widely used as a chemical inducer for *in vitro* differentiation of human mesenchymal stem cells (Setiawan et al., 2022). Our investigation into the dose-dependent effects of BMP4 on hPDLSC differentiation provides critical mechanistic insights with direct relevance to clinical translation. scRNA-seq analyses revealed distinct correlations between endogenous *BMP4* expression and lineage-specific transcription factors. *BMP4* expression correlates inversely with *RUNX2* and positively with *SCX* within osteogenic and tenogenic subpopulations, respectively (Figure 1). Furthermore, trajectory analyses uncovered biphasic expression dynamics of *BMP4*, *RUNX2* and *SCX* during hPDLSC cellular state transitions. Specifically, low *BMP4* expression at the onset of hPDLSC cellular transitions coincided with high expression of both *RUNX2* and *SCX*, whereas high *BMP4* expression during transitions showed an inverse correlation with the expression of these two transcription factors (Figure 2), collectively indicating a dose-dependent regulatory mechanism of BMP4 in hPDLSC differentiation. Indeed, *in vitro* functional assays verified these observations: low-dose BMP4 (10 ng/mL) maintained multipotency by simultaneously upregulating both *RUNX2* and *SCX* (Figure 3), consistent with previous studies showing BMP4’s role in maintaining PDLSC stemness (Liu et al., 2013). Supporting this, manipulating the microenvironment of bone marrow-derived mesenchymal stem cells (BMSCs) with BMP4 can rescue the stemness loss during successive passaging (Chen et al., 2021). Together, these findings suggest that low-dose BMP4 may serve as a conserved regulator of multipotency in adult mesenchymal stem cells. Notably, high-dose BMP4 (100 ng/mL) promoted tenogenic differentiation while suppressing osteogenic commitment (Figure 3). This aligns with our previous study (Li et al., 2013), which showed that overexpression of *BmprIa*, a main receptor of Bmp4 signaling, in mouse cranial neural crest cells impaired odontoblast differentiation and reduced dentin deposition, effectively inhibiting hard tissue formation. This finding parallels our current observation that excessive BMP signaling inhibits osteogenic differentiation (Figure 3). Conversely, genetic reduction of Bmp signaling in *Wnt1Cre;pMes-caBmprIa;BmprIa* ^*F/+*^ mice restored normal odontoblast differentiation (Li et al., 2013). Our previous and current studies that high-concentration BMP4 favors tenogenic differentiation appear to contradict the well-established function of BMP4 in promoting bone formation (Beederman et al., 2013). We speculate that this paradox arises from dose-dependent signaling dynamics. While BMP-2/-7 typically induce osteo/cementoblastic differentiation via Smad1/5/8 phosphorylation and RUNX2 upregulation (Acil et al., 2016; Sacramento et al., 2022), elevated BMP4 concentrations may activate non-canonical pathways (e.g., MAPK/ERK) that preferentially drive fibroblastic/tenogenic phenotypes (Hyun et al., 2017). However, it is important to note that our BMP4 treatments were short-term (1–3 days). Given that SCX + progenitor cells can form bone eminences under regulation of TGFβ and BMP4 signaling (Blitz et al., 2013), future long-term treatment studies are warranted to clarify the osteogenic potential of SCX + progenitor cells. Another crucial finding was the identification of lineage-specific BMP receptor utilization (Figure 4), suggesting that receptor-targeted strategies could selectively modulate differentiation processes. This insight holds promise for guiding biomaterial design, enabling spatial control over differentiation outcomes to match the complex tissue requirement of periodontal regeneration.

Beyond BMP4’s regulatory role, our findings reveal unprecedented heterogeneity within hPDLSC subpopulations, challenging the traditional view of PDLSCs as homogeneous stem cell population and providing crucial insights for therapeutic optimization. Current PDLSC isolation protocols may inadvertently enrich for specific subpopulations while depleting others, potentially compromising therapeutic efficacy. For successful periodontal regeneration, therapeutic strategies must account for subpopulation composition to ensure adequate representation of cells capable of differentiating into all necessary tissue types.

Collectively, our study establishes that BMP4 elicits dose-dependent effects on hPDLSC lineage specification and uncovers previously unprecedented cellular heterogeneity, both of which are critical for refining regenerative therapies. By demonstrating that BMP4 concentration can bias differentiation while preserving multipotency, we provide a practical approach to addressing a fundamental challenge in periodontal regeneration: coordinating differentiation into multiple tissue types within a single intervention. These findings offer promising directions for developing more effective periodontal regenerative therapies for treating chronic periodontitis. Biomaterial-based approaches, such as spatially compartmentalized scaffolds with controlled release kinetics (Park et al., 2020; Rong et al., 2024), or 3D-printed constructs with regionally distinct internal microstructures (Desai et al., 2024), could potentially mimic the dose-dependent effects observed in our study by delivering lower BMP4 concentrations to maintain PDLSC multipotency in certain regions while providing higher concentrations to promote tenogenic differentiation where needed. However, caution must be exercised to avoid potential risks such as ectopic bone formation or heterotopic ossification with excessive BMP4 exposure (Meyers et al., 2019), which could compromise the regeneration of functional periodontal ligament tissue with its requisite fibrous architecture. Strategies to mitigate these risks may include spatial compartmentalization of BMP4 gradients within regenerative constructs (Chen et al., 2020), sequential or temporally controlled delivery protocols (Park et al., 2020), or combination approaches with other signaling factors that balance osteogenic and tenogenic differentiation to achieve proper tissue architecture. These combinatorial strategies are particularly relevant given that PDLSC fate is regulated by a complex network of signaling pathways, including Wnt/β-catenin, MAPK, TGF-β/Smad, BMP, and PI3K/AKT pathways (Zhu and Liang, 2015; Ru et al., 2023; Zhao et al., 2025).

Despite these contributions, several limitations should be acknowledged. First, our *in vitro* studies used fixed BMP4 concentrations for short treatment durations, which may not fully recapitulate the complex, dynamic growth factor milieu *in vivo*. Second, scRNA-seq analysis was performed on PDLSCs from healthy donors; disease-related changes in cellular heterogeneity (e.g., in chronic periodontitis) remain to be investigated. Third, while our inferred receptor utilization model (Figure 4) is supported by previous work on BMPR1A’s role in hard tissue formation (Li et al., 2013) and BMPR1B’s potential role in tenogenic differentiation (Zhang et al., 2019), direct receptor-specific loss-of-function experiments would provide definitive confirmation and represent an important future research direction.

## 4 Materials and methods

### 4.1 Bioinformatics analysis of hPDLSC scRNA-seq data

Publicly available hPDLSC scRNA-seq data (Yang et al., 2024) from third molars of three healthy donors (aged 21–27 were processed and subjected to unsupervised clustering with the standard Seurat (Hao et al., 2024) pipeline and parameters suggested by VECTOR software (Zhang et al., 2020). Since these hPDLSC were obtained from three individual donors, discontinuous clusters from unsupervised clustering were excluded from downstream analyses. Uniform Manifold Approximation and Projection (UMAP) and principal component analysis (PCA) embeddings from the resultant Seurat object were then used by VECTOR to infer developmental directions. To reconstruct hPDLSC differentiation trajectories, cell embeddings and clusters from the resultant Seurat object were converted to a CellDataSet object and ordered in pseudotime using Monocle2 (Qiu et al., 2017) with default parameters. For analysis of cell-cell communication between hPDLSC subpopulations, we employed CellChat (Jin et al., 2021). Gene expression data and cell group information from the resultant Seurat object were used to create a CellChat object for computing communication probability between hPDLSC subpopulations. Incoming BMP signaling to osteogenic/RUNX2+ and tenogenic/SCX + cells was then visualized using CellChat’s internal function netVisual_bubble. Pseudobulk analysis between SCX+ and RUNX2+ hPDLSC cells were performed on raw counts extracted from the processed Seurat object using DESeq2 (Love et al., 2014). Differentially expressed genes (DEGs) were visualized using the EnhancedVolcano package (Blighe et al., 2018). DEGs with p-value <0.05 were considered statistically significant and subjected to functional classification using clusterProfiler (Yu et al., 2012).

### 4.2 Cell isolation, culture and treatments

Human periodontal ligament stem cells (hPDLSCs) were isolated according to a previous study (Schweitzer et al., 2001). Normal third molars (n = 10) were collected at School and Hospital of Stomatology, Fujian Medical University. Briefly, periodontal ligament cells were scraped from third molars and digested with 3 mg/mL collagenase type I (Worthington Biochem) and 4 mg/mL dispase (Roche) for 1 h at 37 °C. Isolated hPDLSCs were then cultured in Dulbecco’s modified Eagle’s medium supplemented with 10% fetal bovine serum, 200 U/mL penicillin, 200 μg/mL streptomycin, and 100X GlutaMAX™ Supplement (all from Gibco). Cells were grown at 37 °C in a humidified atmosphere containing 5% CO_2_. Passages 3-5 of hPDLSCs were used for experiments. After reaching 80%–90% confluence, cells were incubated with BMP4 (R&D Systems), NOG (R&D Systems), or control and BMP4 lentivirus (pNL-BMP4-IRES2-EGFP) at indicated concentrations for specified time periods in complete medium. The medium was changed every other day.

### 4.3 RNA extraction and real time-quantitative PCR (RT-qPCR)

Total RNA was isolated using the Simply P Total RNA Extraction Kit (BioFlux) according to the manufacturer’s instructions. Complementary DNA was reverse-transcribed from total RNA using the PrimeScript RT Reagent Kit (Takara). RT-qPCR was performed in triplicate using SYBR Premix Ex Taq™ (Takara) on a Thermal Cycler Dice™ Real Time System TP800 (Takara). Five independent experiments were performed, and mean expression levels are shown. The data were analyzed with the Thermal Cycler Dice™ Real Time System analysis software (Takara). Data were analyzed using the Thermal Cycler Dice™ Real Time System analysis software (Takara). Gene expression levels were normalized to ACTB and expressed as fold changes relative to control. Primer sequences for all genes are listed in Supplementary Table S1.

### 4.4 Immunofluorescence

For immunofluorescence analysis, hPDLSCs were sub-cultured on glass coverslips in 24-well cell culture dishes. After lentivirus treatment for 96 h, cells were washed with 1× PBS and fixed in 4% paraformaldehyde (PFA) for 10 min at room temperature. After removing PFA and washing twice with PBS containing 0.1% Tween-20, cells were blocked and incubated with SCX primary antibody (Abcam, ab58655, 1:200) for 2 h, followed by incubation with Alexa Fluor-488-conjugated secondary antibody. DAPI (Invitrogen) staining was used to label nuclei according to the manufacturer’s instructions. Images were acquired using a Leica DM 5500B microscope equipped with a Leica DFC 495 camera or a Zeiss LSM700 meta-confocal laser-scanning microscope. To quantify cytoplasmic and nuclear SCX fluorescence intensity, we employed CellProfiler (Stirling et al., 2021). Briefly, nuclei were identified using Global thresholding with the Otsu approach. Whole cells and cytoplasm were then segmented using the Identify Secondary and Tertiary Objects modules. Mean SCX intensity was measured for nuclei and cytoplasm, and nucleus-to-cytoplasm ratios were calculated. Data were exported to Excel for analysis in Prism.

## 5 Conclusion

Our study reveals previously unrecognized cellular heterogeneity in hPDLSCs and demonstrates that BMP4 concentration can bias their differentiation outcomes while preserving multipotency, eliciting dose-dependent effects on hPDLSC lineage specification. These findings provide invaluable insights for refining periodontal regenerative therapies in the treatment of chronic periodontitis.

## Data Availability

The datasets presented in this study can be found in online repositories. The names of the repository/ repositories and accession number(s) can be found below: https://www.ncbi.nlm.nih.gov/geo/query/acc.cgi?acc=GSE227731.

## Abbreviations

PDLSC, Periodontal Ligament Stem Cells.
