# Supplementary material for: BMP4 dose dictates lineage specification bias in human periodontal ligament stem cells
Source: Front Bioeng Biotechnol. 2026 Jan 6;13:1738051. doi: 10.3389/fbioe.2025.1738051 (PMC12816240; doi:10.3389/fbioe.2025.1738051)
Supplement: Supplementary file 1 [file Supplementaryfile1.docx]

Supplementary Material

# 1 Supplementary Figures and Tables

## Supplementary Figures


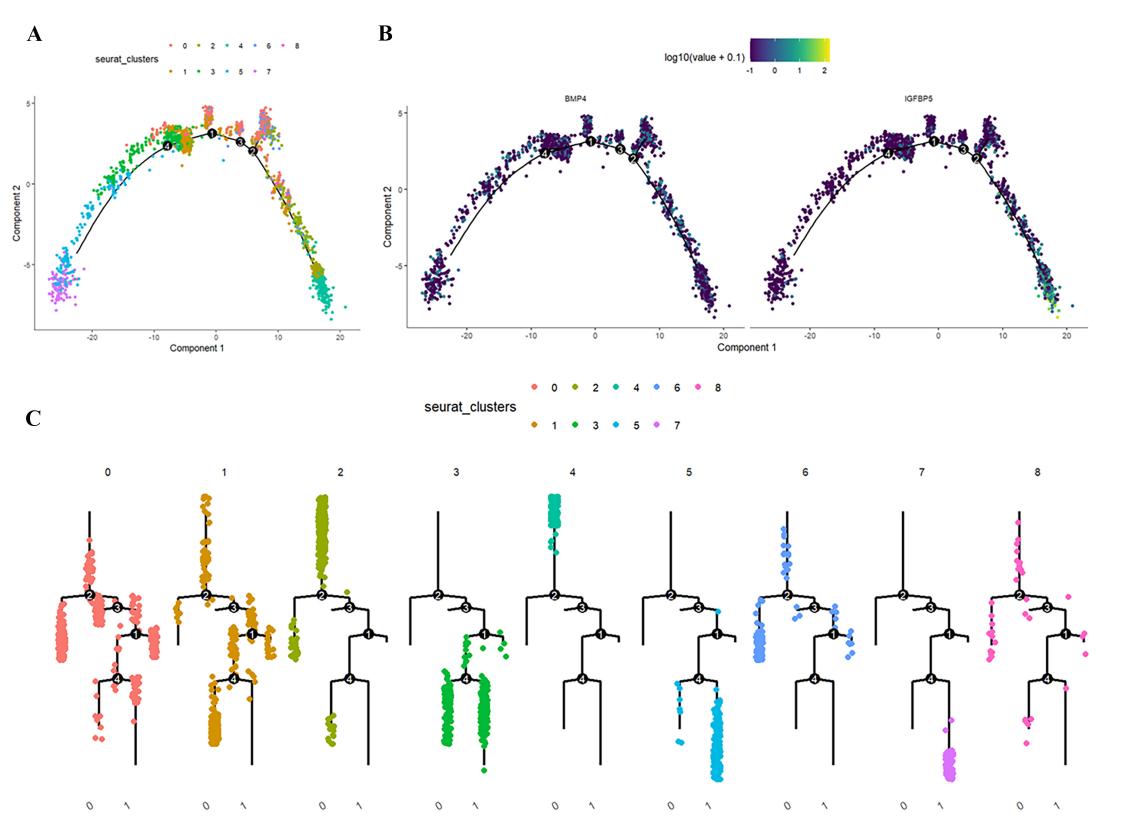


**Supplementary Figure 1.** (A) Distribution of 9 cell subpopulations (clusters 0-8) along the pseudotemporal differentiation trajectory of hPDLSCs. (B) Cell subpopulation distribution (clusters 0-8) at different branch points (number 1-4) during hPDLSC differentiation

## Supplementary tables

Primers for RT-qPCR.

| ACTB | F | 5-TAGTTGCGTTACACCCTTTCTTG-3 |
| --- | --- | --- |
|  | R | 5-TCACCTTCACCGTTCCAGTTT-3 |
| SCX | F | 5-AGAACACCCAGCCCAAACA-3 |
|  | R | 5-TCCTTGCTCAACTTTCTCTGGT-3 |
| RUNX2 | F | 5-TCCACAAGGACAGAGTCAGATT-3 |
|  | R | 5-GCTCAGGTAGGAGGGGTAAGAC-3 |
